# Small cell lung cancer: circulating tumor cell lines and expression of mediators of angiogenesis and coagulation

**DOI:** 10.37349/etat.2023.00139

**Published:** 2023-04-28

**Authors:** Barbara Rath, Adelina Plangger, Lukas Klameth, Maximilian Hochmair, Ernst Ulsperger, Bram Boeckx, Christoph Neumayer, Gerhard Hamilton

**Affiliations:** 1 Institute of Pharmacology, Medical University of Vienna, 1090 Vienna, Austria; 2 Center for Pathophysiology, Infectiology and Immunology, Medical University of Vienna, 1090 Vienna, Austria; 3 Karl Landsteiner Institute of Lung Research and Pulmonary Oncology, Hospital Floridsdorf, 1210 Vienna, Austria; 4 Hospital Horn, 3580 Horn, Austria; 5 Laboratory for Translational Genetics, Department of Human Genetics, University of Leuven, 3580 Leuven, Belgium; 6 Department of Vascular Surgery, Medical University of Vienna, 1090 Vienna, Austria; Università degli Studi della Campania “Luigi Vanvitelli”, Italy

**Keywords:** Small cell lung cancer, circulating tumor cells, tissue factor, vascular endothelial growth factor, coagulation, metastasis

## Abstract

**Aim:**

Coagulation is frequently activated in cancer patients and has been correlated with an unfavorable prognosis. To evaluate whether a putative release of tissue factor (TF) by circulating tumor cells (CTCs) represents a target to impair the dissemination of small cell lung cancer (SCLC), the expression of relevant proteins in a panel of permanent SCLC and SCLC CTC cell lines that have been established at the Medical University of Vienna.

**Methods:**

Five CTC and SCLC lines were analyzed using a TF enzyme-linked immunosorbent assay (ELISA) tests, RNA sequencing, and western blot arrays covering 55 angiogenic mediators. Furthermore, the influence of topotecan and epirubicin as well as hypoxia-like conditions on the expression of these mediators was investigated.

**Results:**

The results demonstrate that the SCLC CTC cell lines express no significant amounts of active TF but thrombospondin-1 (TSP-1), urokinase-type plasminogen activator receptor (uPAR), vascular endothelial-derived growth factor (VEGF) and angiopoietin-2 in two cases. The major difference between the SCLC and SCLC CTC cell lines found was the loss of the expression of angiogenin in the blood-derived CTC lines. Topotecan and epirubicin decreased the expression of VEGF, whereas hypoxia-like conditions upregulated VEGF.

**Conclusions:**

Active TF capable of triggering coagulation seems not to be expressed in SCLC CTC cell lines in significant levels and, thus, CTC-derived TF seems dispensable for dissemination. Nevertheless, all CTC lines form large spheroids, termed tumorospheres, which may become trapped in clots of the microvasculature and extravasate in this supportive microenvironment. The contribution of clotting to the protection and dissemination of CTCs in SCLC may be different from other solid tumors such as breast cancer.

## Introduction

Thrombosis is a common complication of cancer patients but the mechanisms that promote coagulation as well as the fibrinolytic pathway and platelet activation are not clear [1]. Abnormalities *in vitro* coagulation assays are found in more than 90% of patients with cancer, irrespective of their thrombotic status and the occurrence of disseminated intravascular coagulation had a negative effect on the survival [2]. Tissue factor (TF), a 47-kDa membrane-associated glycoprotein, has been identified as a key player in the hemostatic system and cancer progression [3]. The blood coagulation cascade is initiated upon the binding of factor VIIa (FVIIa, activated FVII) to TF, triggering the activation of coagulation zymogens and the formation of fibrin clots [3]. The expression of TF is up-regulated in cancer cells and a soluble form, as well as a splice variant, are present in the circulation [4]. Furthermore, cancer patients have an increased risk of developing thrombosis after treatment with cisplatin-based chemotherapies [5]. Tumor cells can constitutively express TF and release it in form of microparticles as reported for breast, melanoma, and hepatocarcinoma cell lines [6].

Dissemination of tumors is effectuated by circulating tumor cells (CTCs) which detach from primary lesions, intravasate, and generate metastases after having survived in the bloodstream [7]. CTCs expressing both vimentin and TF have been identified in more than 80% of metastatic breast cancer patients; however, a subgroup showed no elevation of plasma TF [8, 9]. Small cell lung cancer (SCLC) patients have high numbers of CTCs which had made it possible to establish several permanent SCLC CTC cell lines at the Medical University of Vienna [10]. These lines were established from patients with metastatic disease and share characteristics with respect to growth factor receptors, interaction with normal cell types, and secretion of cytokines [11, 12]. In the present study, the expression of angiogenic and coagulation-related mediators has been studied employing the proprietary panel of SCLC and SCLC CTC lines.

## Materials and methods

### Cell lines, ethical approval, and reagents

SCLC26A was established at the Medial University of Vienna from a pleural effusion of an SCLC patient before treatment. The following four SCLC cell lines have been supplemented by the Department of Radiation Biology, the Finsen Centre, Copenhagen, Denmark: the permanent cell line GLC16 has been derived from a patient after chemotherapy, DMS153 was established from a liver lesion of a pretreated patient, and NCI-H526 from bone metastasis, respectively, whereas NCI-H417 stems from primary SCLC before treatment. The SCLC CTC cell lines BHGc7, BHGc10, BHGc16, BHGc26, and UHGc5 were established from peripheral blood of SCLC patients with the extended disease after second-line therapy [10–12]. With exception of SCLC26A all other SCLC, lines express achaete-scute homolog 1 (ASCL1) and belong to the SCLC-A subgroup. Blood collection and generation of cell lines were done according to the ethics approval 366/2003 by the Ethics Committee of the Medical University of Vienna, Vienna, Austria. After the successful initiation of the cells, cultivation was performed in RPMI-1640 standard medium (R8758, Sigma-Aldrich, St. Louis, MO, USA) supplemented with 10% fetal bovine serum (S0115, Seromed, Berlin, Germany) and antibiotics (P4333, Sigma-Aldrich, St. Louis, MO, USA). Epirubicin (E9406), topotecan (T2705) and deferoxamine mesylate (DFX, D9533) were obtained from Sigma-Aldrich (St. Louis, MO, USA).

### Angiogenesis factors western blot array

Proteins involved in angiogenesis and coagulation were analyzed using the Angiogenesis Proteome Profiler Array (ARY007, R&D Systems, Minneapolis, MN, USA) according to the manufacturer’s instructions. Experiments were done in duplicate and the different tests were calibrated using the six reference spots included for each individual membrane. A pixel intensity value of 500 corresponds to background levels (pixel intensity of empty positions). Arrays were evaluated using ImageJ (NIH, Bethesda, MD, USA) and Origin 9.1 software (OriginLab, Northampton, MA, USA).

### TF enzyme-linked immunosorbent assay

Cell culture supernatants were assayed using the human coagulation FIII/TF Quantikine enzyme-linked immunosorbent assay (ELISA, R&D systems, Minneapolis, MN, USA). This test has a sensitivity of 2.05 ng/L TF and a measurement range of 7.8–500 ng/L.

### Cytotoxicity assays

Aliquots of 1 × 10^4^ cells in 100 μL medium were distributed to wells of flat-bottom 96-well microtiter plates (TPP, 11311714, Trasadingen Switzerland), and ten 2-fold dilutions of the test compounds were added. Assays were at least performed in triplicate. The plates were incubated for four days under tissue culture conditions (37°C, 5% CO_2_, incubator) and viable cells were detected using a modified 3-(4,5-dimethylthiazol-2-yl)-2,5-diphenyltetrazolium bromide (MTT) assay (EZ4U, Biomedica, Vienna, Austria). Half-maximal drug inhibitory concentration (IC_50_) values were determined using Origin 9.1 software.

### Pretreatment of the CTC cell lines

Cells were incubated in 75 cm^2^ tissue culture flasks in standard medium with the respective concentrations of epirubicin (250 nmol/L), topotecan (200 nmol/L), or DFX (25 μmol/L) for three days. Supernatants were used for the angiogenesis arrays. Additionally, cells were collected, washed with standard medium, counted, and used in MTT viability assays as described.

### RNA sequencing

RNA was extracted using TRIzol (Invitrogen, Carlsbad, CA, USA). Starting from 1 ug total RNA, polyadenylated fragments were isolated, reverse transcribed, and converted into indexed sequencing libraries using the KAPA stranded messenger RNA (mRNA)-Seq kit (Sopachem, Eke, Belgium). The first 51 bases of these libraries were sequenced on an Illumina HiSeq 2500 platform. The raw single-end sequenced reads were aligned to the reference transcriptome and genome using the Bowtie TopHat pipeline (Center of Comutional Biology, Johns Hopkins University, Baltmore, MD, USA). Mapped reads were assigned to ensemble gene IDs by HTSeq and further normalization was performed with the EDASeq package.

### Statistical analysis

Statistical significance was tested by *t*-tests and *P* < 0.05 was regarded as a significant difference using Origin 9.1 software.

## Results

### Angiogenesis array of supernatants of SCLC and SCLC CTC cell lines

**Figure 1.**
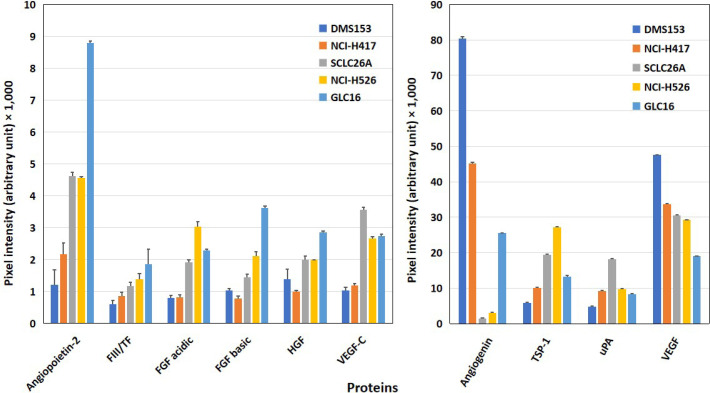
Western blot array results of the expression of angiogenic proteins of SCLC cell lines. Of the 55 angiogenesis array proteins tested, 10 proteins involved in homeostasis are shown in this figure for a panel of SCLC permanent cell lines [mean ± standard deviation (SD)]. The figure is split into proteins with low expression (left) and prominently expressed proteins (right). With exception of angiogenin and VEGF, all other mediators shown are significantly higher expressed in SCLC26A, NCI-H526, and GLC16 cells compared to the DMS153 and NCI-H417 cell lines

Cell lines tested for angiogenetic factors were DMS153, NCI-H417, NCI-H526, GLC16, and primary SCLC line SCLC26A (Figure 1). All cell lines showed expression of angiopoietin-2, thrombospondin-1 (TSP-1), urokinase-type plasminogen activator (uPA), and vascular endothelial growth factor (VEGF). DMS153, NCI-H417, and GLC16 exhibited expression of angiogenin, whereas SCLC26A and NCI-H526 lacked this protein. TF, with exception of DMS153, fibroblast growth factor (FGF) acidic, FGF basic, hepatocyte growth factor (HGF), and VEGF-C were found at low to medium levels in SCLC26A, NCI-H526, and GLC16, respectively.

**Figure 2.**
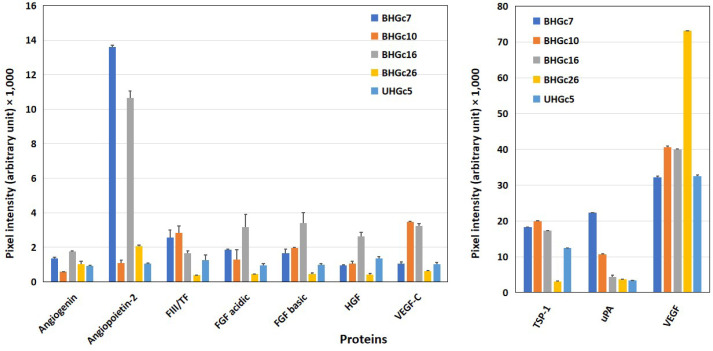
Western blot array results of the expression of angiogenic proteins of SCLC CTC cell lines. Again, 10 of 55 angiogenesis array proteins are shown in this figure for a panel of SCLC CTC permanent cell lines (mean ± SD) with proteins with low expression (left) and prominently expressed proteins (right) separated. As significant differences, high protein levels of angiopoietin-2 in BHGC7 and BHGc16, of TSP-1, with exception of BHGc26 in the other lines, uPA for BHGc7 and VEGF in the SCLC CTC cell lines were found. Furthermore, BHGc16 exhibited significantly elevated FGF variants and HGF as well as VEGF-C. TF levels of BHGc7, BHGc10, and BHGc16 seem to exceed background levels but these data are checked by an ELISA kit with higher sensitivity and specificity in further experiments

Furthermore, angiogenetic proteins were determined in western blot arrays for the 5 SCLC CTC cell lines BHGc7, BHGc10, BHGc16, BHGc26, and UHGc5 (Figure 2). Significant protein expression was found for TSP-1, uPA, and VEGF and two lines for angiopoietin-2, namely BHGc7, and BHGc16. TF was below the detection limit of this western blot array in supernatants of the SCLC CTC lines and angiogenin, FGFs, HGF as well as VEGF-C.

### Effects of treatment with epirubicin or topotecan on expression angiogenic proteins by SCLC lines

**Figure 3.**
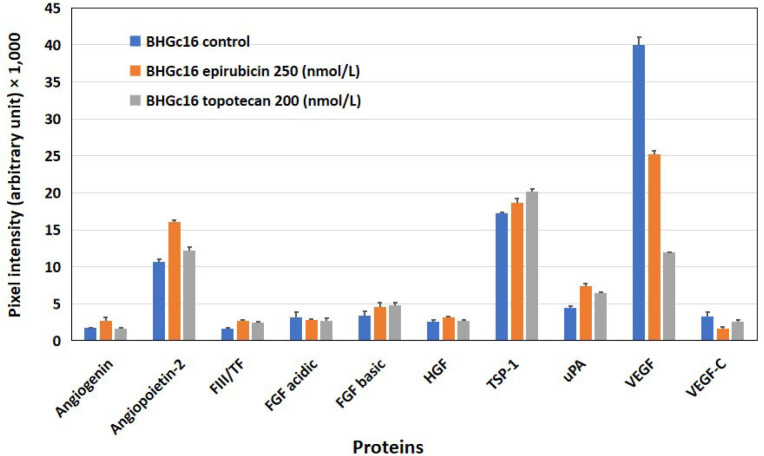
Effects of epirubicin and topotecan on the expression of angiogenic proteins in BHGc16 CTCs. The effects of two chemotherapeutics and hypoxia-like conditions were investigated for BHGc16, BHGc7, and BHGc10, respectively. The figure shows the effects of epirubicin (250 nmol/L) and topotecan (200 nmol/L) on the expression of the 10 angiogenesis-related proteins. BHGc16 reveals significantly increased expression of angiopoietin-2, TSP-1, and uPA in response to both chemotherapeutics, whereas VEGF is markedly reduced (all differences statistically significant against control). Data represent mean ± SD

The expression of angiogenetic proteins of BHGc16 SCLC CTCs was assayed after treatment of the cells with two chemotherapeutics, namely epirubicin, and topotecan, which are clinically administered for second-line treatment of disseminated SCLC (Figure 3). Concentrations of epirubicin and topotecan were selected to provide 80% viability of the tumor cells, as assessed in MTT assays. *In vitro* exposure of the cells to epirubicin resulted in increased expression of angiopoietin-2 and uPA, whereas VEGF showed a marked reduction. Similarly, the application of topotecan resulted in a slight increase in angiopoietin-2 and uPA as well as a still greater reduction of the release of VEGF compared to epirubicin.

### Effects of hypoxia-like conditions on expression of angiogenetic proteins

**Figure 4.**
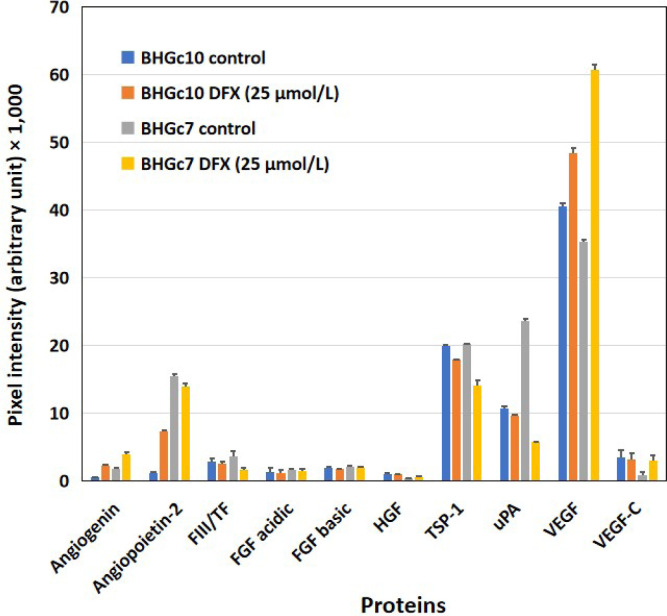
Effects of hypoxia-like conditions on the expression of angiogenic proteins of BHGc10 and BHGc7. The figure demonstrates the influence of DFX-induced hypoxia-like conditions (25 μmol/L DFX, 3 days) on the expression of the 10 angiogenesis array proteins. Angiopoietin-2 and VEGF are significantly induced in BHGc10 CTCs and although VEGF is likewise significantly overexpressed in BHGc7, the protein levels of TSP-1 and uPA are reduced in this case (all differences statistically significant against control, except for uPA and BHGc10). The level of TF is unchanged in BHGc10 and exhibits a minor but significant reduction in BHGc7 in response to DFX. Data represent mean ± SD

A hypoxia-like state was induced in BHGC10 and BHGc7 by preincubation of the cells with DFX. The concentration of DFX was selected to provide 80% viability of the tumor cells, as assessed in MTT assays. Angiogenin was increasingly expressed in both CTC lines tested and angiopoietin-2 in BHGc10, respectively. uPA was markedly decreased in BHGc7, whereas the expression of VEGF showed a significant increase (Figure 4). TF is expected to show increased expression under hypoxic conditions but this experiment actually demonstrates a reduced expression with an 11% reduction for BHGc10 and a 44% reduction for BHGc7, respectively.

### TF ELISA assay

**Figure 5.**
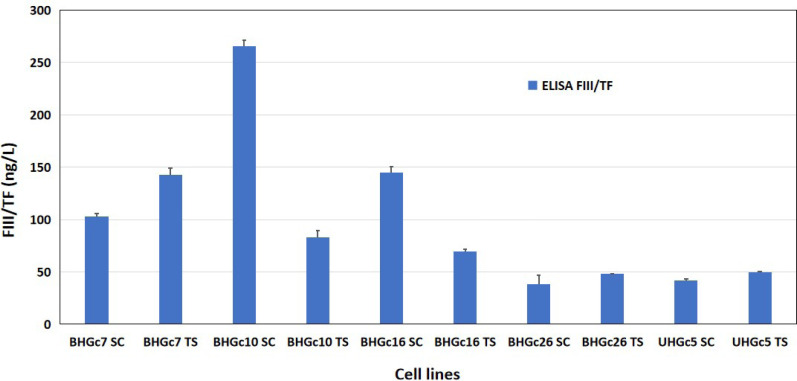
Assay of TF in supernatants of SCLC CTC single cells and tumorospheres. Concentrations of TF were determined using an ELISA assay. For the SCLC CTC lines, single-cell cultures were compared to the same cells in form of tumorospheres. Data represent mean ± SD. The TF concentrations were measured parallel to the data found in the ARY007 array for the CTC lines. The expression of TF in tumorospheres compared to single-cell suspensions is significantly different except for the BHGc26 pair

Conditioned media (CM) of all SCLC CTC cell lines preincubated for 3 days were checked in an ELISA assay with a sensitivity of 2.05 ng/L. The cell lines were tested either in form of single cells or as compact tumorospheres and yielded TF values ranging from 38.3 ng/L to 265.22 ng/L (Figure 5). The differences between single cells and tumorospheres are statistically significant for all BHGcX pairs, except for BHGc26. For comparison, the mean TF concentration for the pleura-derived SCLC cell lines was 41.8 ± 8.0 ng/L (range: 31.5 ng/L–51.3 ng/L) and the mean TF for 7 pleura-derived NSCLC lines was 3,832 ± 2,650 ng/L (range 1,105.4 ng/L–8,860.5 ng/L; not shown), respectively.

### Transcription of mediators of angiogenesis in SCLC26A and SCLC CTC

RNA was isolated from SCLC lines and transcripts was evaluated by next-generation sequencing (NGS). The number of reads was normalized between the individual cell lines of the panel. The results show that HGF, uPA, FGF basic, and TSP-1 (with exception of BHGC7) are expressed in the SCLC CTC lines (Table 1). TF/coagulation FIII shows very low expression in all SCLC/SCLC CTC lines tested. Angiopoietin-2 RNA is expressed at low levels except in BHGc7 and BHGc26. VEGF is markedly expressed in SCLC26A and all SCLC CTC lines.

**Table 1.** RNA expression of angiogenesis factors

**Genes identity**	**SCLC26A**	**BHGc7**	**BHGc10**	**BHGc16**	**BHGc26**	**UHGc5**
Angiopoietin-2 ENSG00000091879	4.67	192.75	16	35	204	7.74
FIII/TF ENSG00000117525	17.12	71.74	50	214	152	57.59
FGF basic ENSG00000138685	38.91	42.03	28	92	20	4.67
HGF ENSG00000019991	0	0	2	4	0	0
Thrombospondin 1 ENSG00000137801	123.35	498.55	7	23	33	6.61
uPA ENSG00000122861	63.42	21.01	6	4	20	0
VEGF ENSG00000112715	2,240.47	3,188.41	3,791	2,019	3,580	1,498.05

The Table 1 shows the number of normalized reads in NGS of mRNA preparations of SCLC26A and the SCLC CTC lines for selected mediators (SD < 14% for duplicate determinations). Transcripts are identified by the gene annotation in Ensembl. Housekeeping gene *GDPDH ENSG00000111640* as standard.

## Discussion

In cancer patients, TF expression has been correlated with tumor grade, increased vascular density, and a worse prognosis [13]. TF promotes activation of FVII to FVIIa, binding of zymogen FX and FIX eventually resulting in coagulation [7]. TF expression in metastatic cancer cells may be upregulated due to inflammation and hypoxia [7, 8, 14]. There is a strong correlation between TF expression, VEGF production, and increased tumor angiogenesis as well as the aggressiveness of pancreas and bladder cancer [15, 16]. In the initial phases of metastasis, fibrin clots and intravascular recruitment of platelets by thrombin seem to be important for the survival of the adherent tumor cells [17]. However, the use of anticoagulants in cancer patients for putative prolongation of survival holds a risk for bleeding [18]. In fresh frozen sections of SCLC tissues, tumor cells stained positively for TF and fibrin at the host-tumor interface but uPA was absent [19]. NSCLC cells express high amounts of TF, while SCLC expressed none or low TF, with exception of an aberrant form of TF in adherent NCI-H69 [20]. Furthermore, a screen for TF-dependent coagulation in SCLC24H, 86M1, NCI-H60, NCI-H69, NCI-H82, NCI-H526, NCI-H510, NCI-H146, NCI-N592, NCI-H841 and DMS-79 SCLC cell lines proved negative [21]. These reports are in line with the present results demonstrating very low or absent expression of TF in SCLC and SCLC CTC lines.

In circulation, only a tiny fraction of cells survive and eventually form metastases after becoming trapped in the microvasculature, possibly in aggregates with platelets [22]. Small clusters of CTCs consisting of several tumor cells are rare but have a higher metastatic potential [23]. The proprietary panel of permanent CTC SCLC lines was employed to study angiogenesis and coagulation effectors. The present results indicate that in SCLC metastasis seems not to be promoted by coagulation-competent CTCs. TF is present at low levels in good agreement with its low protein expression in SCLC CTC cell lines, similar to quantities of angiogenin, FGF, and HGF. In diverse tumor cells that show significant expression of TF, levels equivalent to the expression of VEGF are detectable [24].

Unfortunately, it is not clear which kind of TF is recognized by the commercial ELISA assays—either alternatively spliced TF, microvesicle-associated TF, or degraded TF fragments [25]. The Quantikine^®^ kit has been used for the measurement of TF antigen in plasma from healthy controls and patients with cancer, reporting median levels of TF antigen of 39.1 ng/L (range 16.8 ng/L–87.3 ng/L) for healthy individuals, 47.2 ng/L for patients with cancer without thrombosis, and 56.0 ng/L (range 37.6 ng/L–318.7 ng/L) for patients who developed thrombosis [26]. This assay detects TF in cell lysates and supernatants of TF-expressing cell lines but the low amounts of inactive TF species detected in ELISA assays seem not to be capable of initiating the FVII cascade. Furthermore, the SCLC CTC cells exposed to hypoxia-like conditions fail to exhibit the typical increase of TF, again indicating a lack of expression of active TF species. The BHGC10 CTC line may be an exception and is distinguished by exhibiting primary resistance to cisplatin. Thus, SCLC CTC lines seem to lack expression of functional TF and are not expected to contribute to TF to a significant extent for the dissemination of these cells. Determination of RNA levels by NGS proved the low-level transcription of TF in SCLC CTC cell lines. The angiogenesis protein arrays reveal the high expression of TSP-1, uPA, and VEGF by SCLC and SCLC CTC cell lines. The remaining expression of angiogenin in SCLC cell lines is not any longer observed in the SCLC CTCs. Tumor cell-derived TSP-1 increases uPA, uPA receptor (uPAR), and plasmin activity resulting in decreased tumor cell adhesion and increased cell invasion [27]. VEGF is a key angiogenic factor mediating neovascularization that has been shown to upregulate uPA but despite high expression of VEGF in SCLC, antiangiogenic therapeutics were not effective in prolonging the survival of the patients [28].

An SCLC CTC cell line, namely BHGC16, was precultured with epirubicin and topotecan, which are typically administered for the second-line treatment of metastatic SCLC [29]. Both drugs slightly increased the expression of angiopoietin-2, TSP-1, and uPA, whereas the expression of VEGF was reduced by epirubicin and to a still larger extent by topotecan. SCLC CTC lines BHGc10 and BHGc7 were cultured in a medium supplemented with the hypoxia-mimetic agent DFX resulting in increased expression of angiogenic VEGF [30].

**Figure 6.**
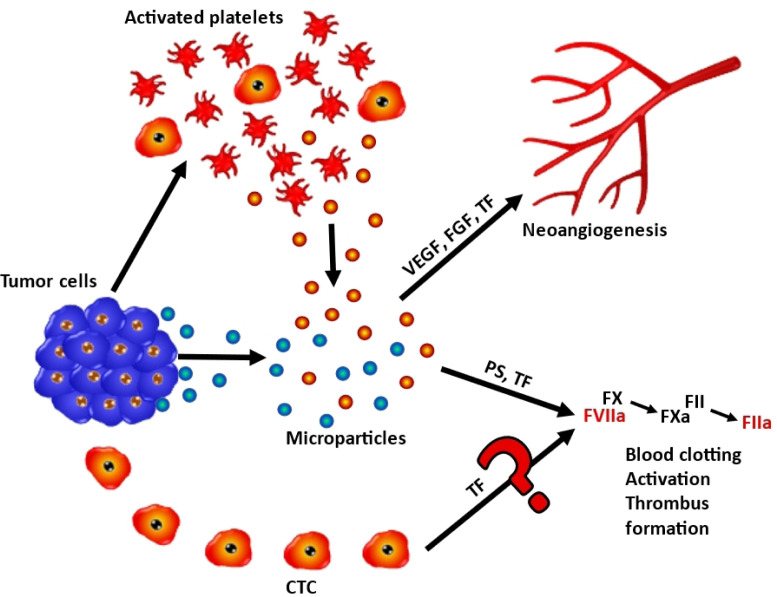
Schematic representation of the role of FIII/TF in coagulation of SCLC and SCLC CTCs. Tumor cells and tumor-activated platelets may release particles containing FIII/TF and other mediators that eventually promote neoangiogensis or trigger the coagulation cascade. Coagulation is initiated by FIII/TF and phosphatidylserine (PS) via FX/FVIIa and FXa followed by activation of FII toFIIa, resulting in blood clotting and trhombus formation. The putative coagulation initiation by CTC-derived release of FIII/TF seems to play no significant role according to the present results

In conclusion, the thromboembolic disease is observed in 6.8% and 11.5% of SCLC patients and is associated with reduced survival. Although TF plays an essential role in coagulation and other functions, SCLC CTCs seem to lack expression of functional TF and the expression of forms of TF in these cells is not inducible by hypoxia-like conditions. Therefore, venous thromboembolism events (VTEs) observed in a minority of SCLC patients are most likely initiated via microparticles released by primary tumors and platelets but not via a contribution of TF by CTCs (Figure 6). Treatment by anticoagulants in lung cancer patients is recommended to be individualized, according to tumor stage and serum levels of D-dimer [31]. Furthermore, the proprietary CTC lines seem to be representative of viable and truly metastasis-inducing cells that are known to account for a very minute fraction of all CTCs. Reports describing a correlation between VTEs and CTC numbers may be linked to larger and more advanced tumors and metastases in patients exhibiting increased numbers of CTCs but may not prove a direct causative role of CTCs.

## Abbreviations

CTCs: circulating tumor cells
DFX: deferoxamine mesylate
ELISA: enzyme-linked immunosorbent assay
FGF: fibroblast growth factor
FVIIa: factor VIIa
HGF: hepatocyte growth factor
MTT: 3-(4,5-dimethylthiazol-2-yl)-2,5-diphenyltetrazolium bromide
SCLC: small cell lung cancer
SD: standard deviation
TF: tissue factor
TSP-1: thrombospondin-1
uPA: urokinase-type plasminogen activator
VEGF: vascular endothelial growth factor

## Declarations

### Copyright

© The Author(s) 2023.
